# The impact of traumatic injury on the respiratory system; a narrative review of injury-associated and clinically-induced mechanisms of trauma-associated pneumonia

**DOI:** 10.3389/fmed.2026.1807707

**Published:** 2026-04-22

**Authors:** Fiona Howroyd, Jonathan Weblin, Fang Gao Smith, Niharika A. Duggal, Zubair Ahmed

**Affiliations:** 1 University Hospitals Birmingham NHS Foundation Trust, Birmingham, United Kingdom; 2 Department of Inflammation and Ageing, School of Infection, Inflammation and Immunology, College of Medicine and Health, University of Birmingham, Birmingham, United Kingdom

**Keywords:** hospital-acquired infections, immune system, injury, pneumonia, respiratory complications, trauma

## Abstract

Pneumonia is a common complication after major trauma, affecting approximately one third of all traumatic injury victims. The pathophysiology of trauma associated pneumonia (TAP) is complex, with numerous, injury-associated mechanisms and clinically-induced risk factors, making early detection and precise diagnosis challenging. These features are coupled with extensive immune modulation and profound inflammation, which occur simultaneously after traumatic injury. When TAP occurs, it is associated with poor patient outcomes, including increased mortality rates, longer intensive care unit and hospital admissions and increased likelihood to be discharged to an ongoing care or rehabilitation facility. However, with rising prevalence of antibiotic resistance and multi-drug resistant strains of bacteria, the management of TAP is becoming increasingly complex. With profound effects upon patient recovery, long-term outcomes and healthcare associated costs, there is urgent need for increased understanding and awareness of TAP. In this narrative review we aim to deconstruct normal lung physiology, to understand the direct impact of major trauma upon the respiratory system. Specifically, we examine how major trauma, across a spectrum of injury subtypes, influences immune responses, ventilatory mechanics, neuromuscular control of breathing, airway protection, and brain–lung interactions, and how these processes contribute to the development of TAP. Finally, we highlight the diagnostic limitations of current clinical criteria and explore the emerging potential of artificial intelligence and machine learning to synthesise complex, heterogeneous data for the early and precise prediction of TAP.

## 1 Introduction

Major trauma can be defined as a heterogenous group of life-threatening or life-changing injuries (1, 2). Although there have been significant improvements in trauma care over the past decade, major trauma remains a significant public health issue and is one of the leading causes of death and disability worldwide (3). Accounting for approximately 10% of all global deaths, major trauma is the third leading cause of mortality in all age groups and the most common cause in those below the age of 40 (3, 4). Recent enhancements in pre-hospital care and major haemorrhage control mean more victims are surviving the primary insult of traumatic injury (5, 6). However, “late deaths” in the days to weeks following acute injury remains prevalent due to the risk of secondary, infective complications (7–9).

Health-care associated infections are common after major trauma and account for 80% of the late deaths that occur in the proceeding days to weeks after injury (7–11). A highly prevalent nosocomial infection after injury is pneumonia, affecting approximately one third of all major trauma patients (12, 13). Hospital acquired pneumonia is an acute, lower respiratory infection of the pulmonary parenchyma that occurs after 48 h of hospital admission (14). Outcomes associated with pneumonia after major trauma are universally poor, characterised by increased mortality, prolonged intensive care unit (ICU) and hospital lengths of stay, and an elevated likelihood of discharge to post-acute care or rehabilitation facilities, rather than directly home (15, 16). Although perhaps previously seen as an inevitable and unavoidable consequence after injury, in recent years there has been increased recognition of the unique circumstances of pneumonia induced by major trauma, subsequently termed as trauma associated pneumonia (TAP) (17).

**Figure 1:**
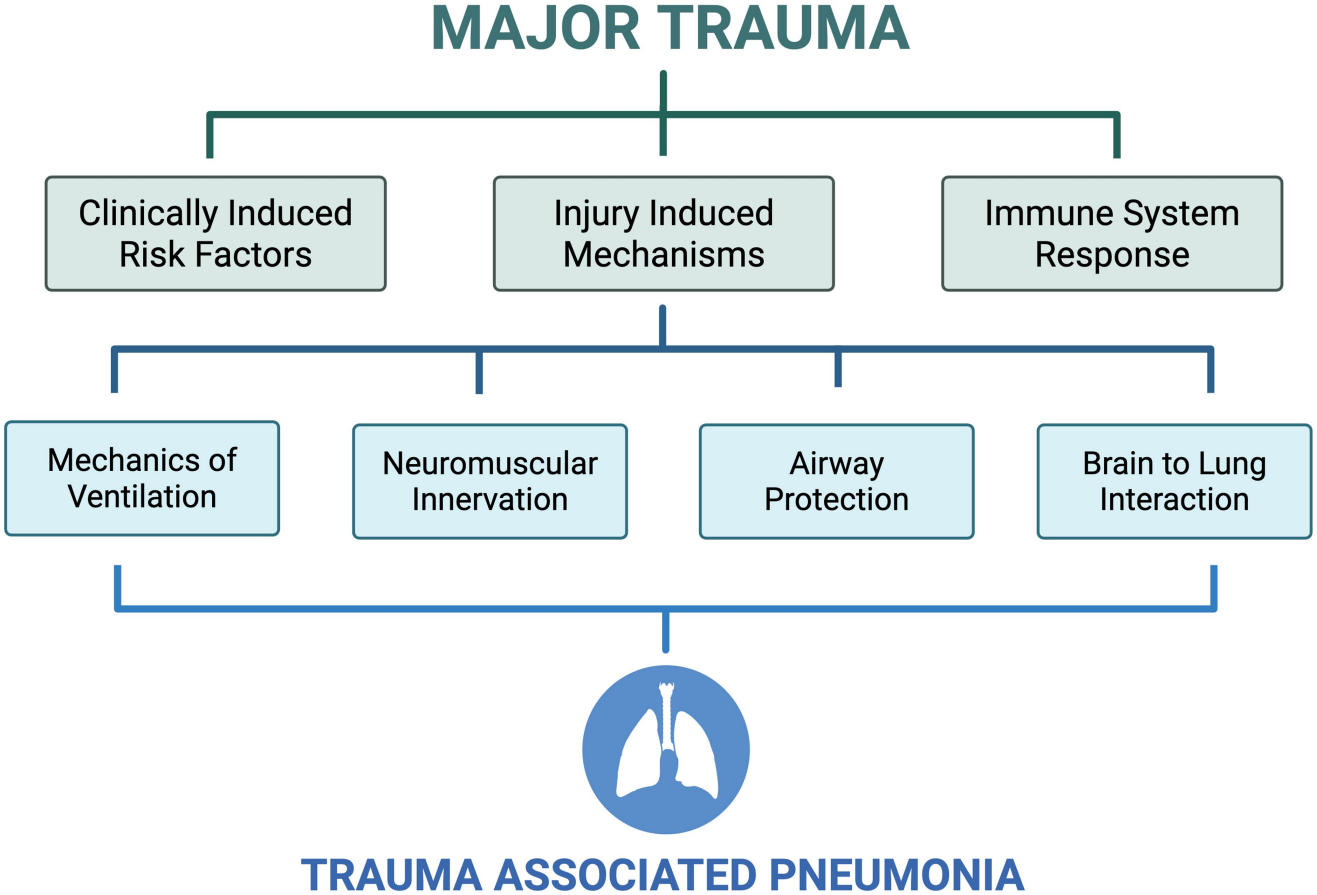
An overview of trauma associated pneumonia. Clinical, injury, and immune induced risk factors and mechanisms after major trauma, influencing normal lung physiology including mechanics of ventilation, neuromuscular innervation of breathing, airway protection, and brain to lung interaction, contributing to trauma associated pneumonia. Created in BioRender.

The pathophysiology of TAP is complex with numerous underlying injury-induced mechanisms which directly impact the respiratory system (17, 18). TAP encompasses heterogenous infections with vast clinical presentations and predisposing risk factors, therefore making precise and early diagnosis challenging (14, 19). Furthermore, the rising prevalence of antibiotic resistance and multi-drug resistant strains of bacteria, means that the early detection and management of TAP in highly complex clinical environments, such as in the trauma ICU, is becoming increasingly challenging. Due to the distinctive injury-associated and clinically-induced risk factors (such as sedation, mechanical ventilation and immobilisation), in addition to the unique and complex immune and inflammatory modulation that occurs after injury, there is a need for detailed understanding of TAP in order to improve diagnosis, management and patient outcomes (20). In this narrative review we aim to explore how major trauma, including a breadth of injury sub-types, affects the normal lung physiology and contributes to infection and respiratory compromise. We aim to describe how major trauma influences the immune system, ventilation mechanics, neuromuscular control of breathing, airway protection, and brain-lung interaction and how this contributes to the development of TAP (Figure 1). Finally, we will discuss the diagnostic challenges of TAP and highlight potential directions for future research.

## 2 The immune response to traumatic injury

**Figure 2:**
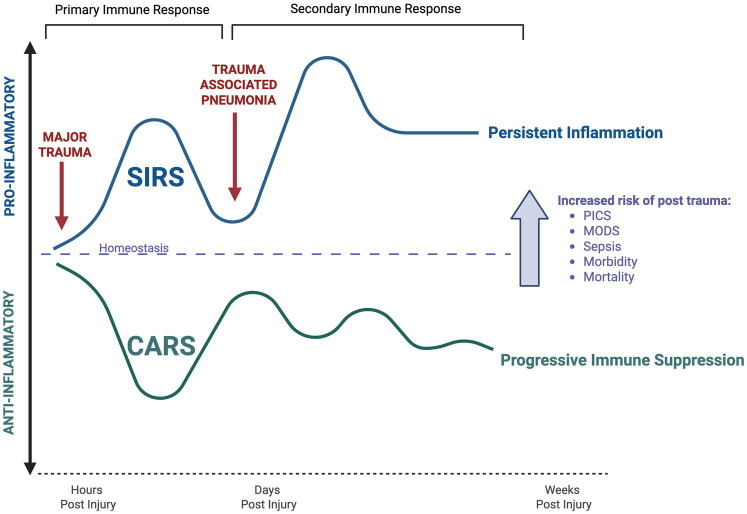
The immune responses to major trauma. Major trauma leads to a primary immune response including pro-inflammatory “Systemic Inflammatory Response Syndrome” (SIRS) and anti-inflammatory “Compensatory Anti-Inflammatory Response Syndrome” (CARS) within hours of injury. This increases the risk of hospital-acquired infections, including trauma associated pneumonia within the proceeding days post injury. This leads to a secondary immune response, featuring persistent SIRS and CARS, leading to increased risk of long-term adverse outcomes, including persistent inflammation-immunosuppression and catabolism syndrome (PICS), multi-organ dysfunction syndrome (MODS), sepsis, morbidity, and mortality. Adapted from Mira et al. (36). Created in BioRender.

Traumatic injury triggers a rapid and complex immune system response, characterised by systemic inflammation and immune activation, alongside compensatory immune suppression (18, 21–23). The immune system responds rapidly to the tissue damage and blood loss induced by injury, leading to profound inflammation within 30 min of trauma, termed systemic inflammatory response syndrome (SIRS) (18, 24). This hyperinflammatory systemic inflammatory response is associated with a symbiotic, counterregulatory, anti-inflammatory and immune suppressive response, termed compensatory anti-inflammatory response syndrome (CARS) (18). This dynamic immune response manifests across numerous innate and adaptive cellular phenotypes and functions, causing disruption of the normal immune homeostasis and thus increased susceptibility to infection (21–25). Some of the key features are summarised below and will be discussed in further detail in relation to specific injury sub-types (Figure 2).

Tissue injury releases antigens and mediators that are recognised by alarmins, termed as damage associated molecular patterns (DAMPs) (22). DAMPs are a heterogeneous group of endogenous danger-signal molecules, including lipids, proteins and deoxyribonucleic acid (DNA), which are systemically released immediately after trauma into the extracellular space (26). Pattern recognition receptors, such as Toll-like receptors, detect DAMPs and also bind to exogenous antigens called pathogen-associated molecular pattern molecules (PAMPs), in order to activate and prime the innate immune system (22). DAMPs and PAMPs play a pivotal role in the systemic inflammatory response after trauma, as they trigger an extensive immune cascade and activate an excessive pro-inflammatory phenotype (22, 26). Consequently, the massive systemic release of DAMPs post-injury elevates traditional acute-phase reactants, such as C-reactive protein (CRP) and procalcitonin, significantly limiting their diagnostic utility for differentiating sterile inflammation from early bacterial infiltration in TAP.

Damage associated molecular patterns (DAMPs) activate neutrophils, monocytes and platelets in addition to complement which generates anaphylatoxins and inflammatory mediators (18). The inflammatory cascade is characterised by rapid release of cytokines, including tumour necrosis factor (TNF), interleukin (IL)-1a, IL-1B, IL-8 and IL-6 within minutes to hours of injury (22, 24, 27). Inflammasomes and toll-like receptors activated by DAMPS also cause macrophages to become hyper-reactive, causing a “second-hit” innate immune system response through excessive inflammatory cytokine production (22, 28). Elevated levels of proinflammatory cytokines further amplifies neutrophil activity (22). The disproportionate activation of neutrophils drives their migrate across damaged endothelium and is associated with dysregulated phagocytosis, generation of reactive oxygen species and formation of neutrophil extracellular traps (18, 29). Reactive oxygen species exacerbates a vicious cycle of inflammation, as hyperactive neutrophils produced excessive inflammatory cytokines and further reactive oxygen species, exacerbating localised lung injury (18, 30). Excessive neutrophil extracellular traps release also contributes to tissue damage and inflammation (31). Despite this heightened pulmonary immune response, systemic immune failure occurs, with increased systemic bacterial dissemination, persistent systemic inflammatory response and cell-mediated tissue damage (24, 31). Super imposed infection introduces additional exposure to pathogen-associated molecular patterns, further stimulating the heightened immune system, leading to a vicious cycle of continued inflammation and immune activation (24).

In addition to their role in the preliminary systemic inflammatory response, damage associated molecular patterns also exert immune suppressive properties (25, 26, 32). Subsequently, alongside the extensive systemic inflammatory response after trauma, there is an opposing, compensatory anti-inflammatory response (CARS), aimed at restoring immune homeostasis (22). Key features include increased expression of anti-inflammatory cytokines such as IL-10 and antagonists such as IL-1RA (33, 34). Compensatory anti-inflammatory response syndrome is largely mediated by the adaptive immune system; with reduced toll-like receptor reactivity, impaired T cell activation and proliferation and diminished T helper 1 (Th1) responses (22, 24). In parallel, macrophage paralysis occurs alongside increased apoptosis of lymphocytes and dendritic cells. These cells subsequently display functional defects in antigen presentation to T cells, further contributing to impaired bacterial clearance (33). This immunosuppressive response subsequently increases the risk of infections, including pneumonia, and is closely related with multi-organ dysfunction syndrome (35).

This complex and co-dependent inflammatory and anti-inflammatory response can result in protracted immune dysregulation, termed persistent inflammation-immunosuppression and catabolism syndrome (PICS), resulting in long-term risk of morbidity and mortality (35, 36). Importantly, this immune dysregulation is not unique to traumatic injury and also arises during surgical trauma and critical illness (33). Furthermore, it has profound and widespread effects on numerous bodily systems and imposes risk for multiple other pathologies; including sepsis, multi-organ dysfunction syndrome, wound infections and acute respiratory distress syndrome (33, 35, 37–39). Although the immune system dysregulation after trauma offer a logical explanation for the risk of pneumonia, the specific mechanisms of TAP remain incompletely defined. However, with this underpinning, we will next discuss the specific consequences of major trauma upon respiratory physiology and explore in detail the risks and mechanisms of TAP.

## 3 Mechanics of ventilation

**Figure 3:**
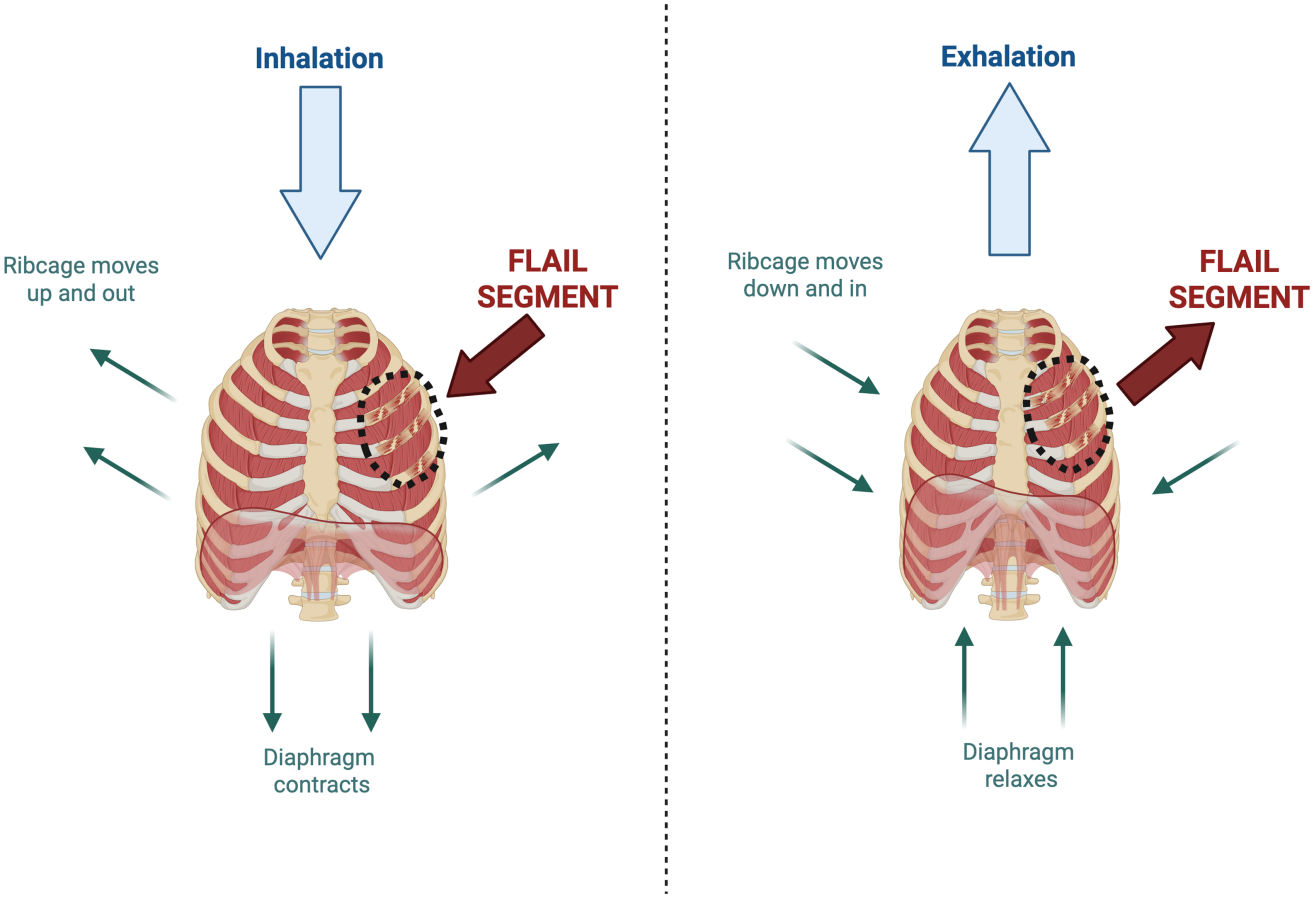
The mechanics of inspiration and expiration and the impact of thoracic trauma. During inspiration, the diaphragm contracts and the ribcage moves up and out, increasing the thoracic volume to allow airflow into the lungs. During exhalation, there is relaxation of the diaphragm, leading to passive recoil of the ribcage and airflow out of the lungs. Thoracic trauma drastically impacts the chest wall mechanics of breathing. Specifically, in the event of a flail segment, there is paradoxical chest wall movement; whereby the flail segment moves inward during inspiration and outward during expiration, independently to the rest of the ribcage. Created in BioRender.

Ventilation is the process of air moving in (inhalation) and out (exhalation) of the lungs, for gas exchange to occur. Inhalation is an active process, whereby the diaphragm contracts to move the ribcage moves up and out and increase the volume of the thoracic cavity. As thoracic volume increases, intrathoracic pressure falls below atmospheric pressure, creating a pressure gradient that allows air to flow from areas of higher pressure into the lungs. Exhalation is a passive process when, due to the relaxation of the diaphragm, there is elastic recoil of tissues to reduce the intrathoracic volume. This subsequently increases the intrathoracic pressure, allowing air to be exhaled out of the lungs. The integrity of the ribcage is therefore paramount to the mechanics of ventilation (Figure 3).

**Figure 4:**
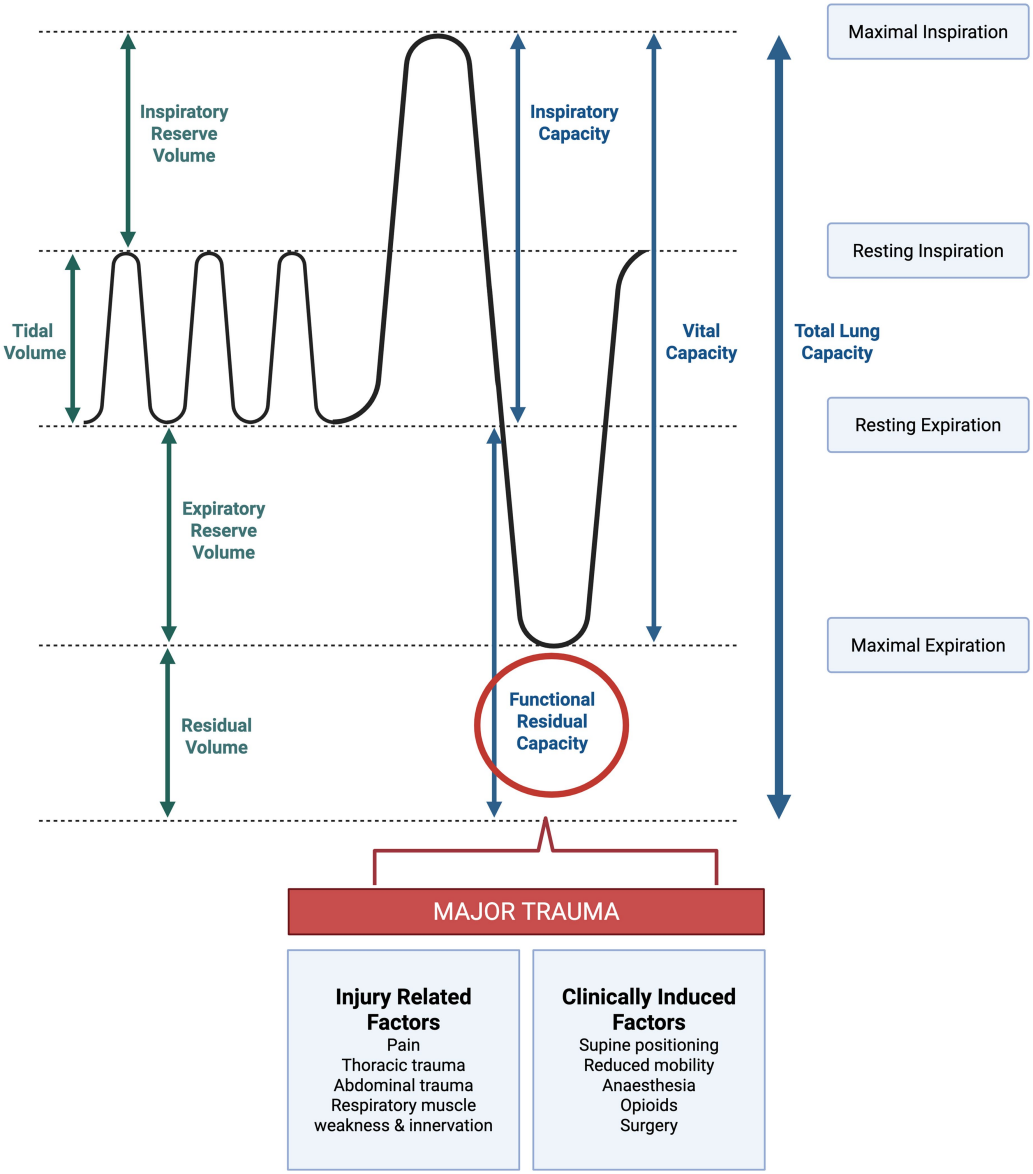
Lung volumes and the effect of major trauma. Lung volumes and lung capacities at resting and maximal inspiration and expiration. Injury related factors and clinically induced factors that influence functional residual capacity after major trauma. Created in BioRender.

Major trauma frequently involves serious injury to the thorax, occurring in approximately 60% of all patients with polytrauma and accounting for a quarter of all trauma-associated deaths (4). Thoracic injuries, particularly multiple rib fractures, disrupt normal respiratory mechanics, increasing pneumonia risk four-fold compared to patients without such injuries (17, 40, 41). In blunt chest trauma, such as road traffic collisions, the thorax absorbs extreme acceleration-deceleration forces and direct impact, causing rib fractures (4). Rib fractures following blunt chest trauma are common, affecting appropriately 10% of all hospitalised trauma patients (42). Due to the location of the neurovascular bundle in the costal groove of each rib, rib fractures result in severe pain, leading to hypoventilation and reduced lung volumes (4, 43). In addition, rib fractures impair the normal chest wall mechanics, decreasing functional residual capacity, forced vital capacity and lung compliance, which promotes sputum retention, atelectasis and infection (40, 41, 43, 44) (Figure 4).

Thoracic trauma also damages lung parenchyma through direct or acceleration-deceleration forces, disrupting the alveolar-capillary membrane, causing pulmonary haemorrhage and oedema, presenting as pulmonary contusions or lacerations (44). These injuries reduce surfactant production, further lowering lung compliance and causing atelectasis (45). Traumatic forces trigger a localised inflammatory response, with increased inflammatory cytokine and coagulatory protein expression (46). The release of inflammatory mediators is also exacerbated by the proceeding hypoxia and release of oxygen free radicals (45). Polymorphonuclear leukocytes and neutrophils infiltrate the area and tissue macrophages are activated, leading to increased reactive oxygen species production, necrosis and apoptosis within the alveolar epithelium (30, 45). Collectively, this localised response impairs gas exchange, reduces bacterial clearance and increases susceptibility to infection (47).

In severe cases of thoracic trauma, a flail chest may be present; whereby three or more adjacent ribs are fractured in two or more places (48). A flail chest is associated with poor patient outcomes including increased risk of pneumonia and mortality (48, 49). Rib fractures with flail cause paradoxical chest wall movement; whereby the flail segment moves inward during inspiration and outward during expiration, independently to the rest of the chest wall (Figure 3). This occurs due to the effect of negative intrapleural pressure and contraction of the diaphragm and respiratory muscles acting on the detached segment (45). These altered mechanics of breathing cause respiratory impairment and reduced functional residual capacity, with a third of patients with flail chest developing pneumonia (49).

In addition to injury-induced alterations of chest wall mechanics, lung volumes and functional residual capacity may also be impeded by the effects of supine positioning during recovery from traumatic injury. Despite the known risk factors associated with bedrest, immobilisation and strict supine positioning is often necessary after complex traumatic injuries, such as following pelvic or spinal fractures, to enable bone and tissue healing (50, 51). During periods of intubation, sedation and mechanical ventilation in the ICU, supine positioning in bed is almost inevitable (52). Delays in clinical or radiological spinal clearance, or the wait for definitive surgical fixation, inherently prolong obligatory supine positioning. This geometrically compounds the risk of secretion retention and micro-aspiration during the most vulnerable acute phases of recovery. In the supine posture, functional residual capacity decreases due to alveolar closure in the dependent lung zones, leading to atelectasis (53). During periods of bed rest and immobility, mucociliary action is also dampened, further contributing to the risk of secretion accumulation and atelectasis (53). Furthermore, there is increased risk of aspiration of gastric content in the supine posture, particularly during periods of enteral nutrition feeding, further exacerbating the risk of pneumonia (14, 53). In the critically ill, there is a threefold reduction in the incidence of pneumonia in patients positioned in the semi recumbent posture, compared to those fully supine (14, 54, 55). Interestingly, studies of early rehabilitation in the trauma ICU have suggested reduced incidence rates of pneumonia with enhanced mobilisation, perhaps suggesting the benefit of upright postures, increasing lung capacity and neuromuscular activation in response to exercise (56).

## 4 Neuromuscular innervation of breathing

The primary muscle responsible for ventilation is the diaphragm. The diaphragm is a dome-shaped musculotendinous sheet that sits at the inferior aspect of the ribcage, separating the thoracic and abdominal cavities by attaching to the xiphoid process of the sternum, internal surfaces of ribs 7 to 12 and the vertebral bodies and intervertebral discs of lumbar spine level (L)1 to L3. Injuries involving the thorax, abdomen or spine can subsequently impact the diaphragm due to its vast surface area and muscular attachments. However, traumatic injury to the diaphragm itself is rare, occurring in less than 4% of all traumatic injuries (57). Nonetheless, when diaphragm injury occurs, it is associated with poor outcomes and high mortality rates, due to its crucial role in ventilation and association with devastating injury (57). Diaphragm injury may occur due to direct penetrating trauma such as stab or gunshot wound, or in very rare cases due to blunt chest wall trauma or blast injury, leading to burst defect of the diaphragm due to extreme increases in intrathoracic pressure (57, 58). Rupture can lead to abdominal content herniation into the intrathoracic cavity, causing atelectasis and respiratory distress (59). Due to its anatomical location, diaphragm injury often coincides with abdominal, thoracic and visceral injury, including vital organs. As such, surgical management of diaphragmatic ruptures are often completed alongside laparotomy or thoracotomy for accompanying injuries; with extensive, complex, surgical procedures commonly associated with post-operative complications such as pneumonia (59).

Motor innervation to the diaphragm is supplied by the phrenic nerve, which originates from nerve roots of cervical spine level (C)3 to C5 of the cervical plexus of the spinal cord (60). Spinal cord injury (SCI) to the upper cervical spine subsequently disturbs the phrenic motor impulses, resulting in paralysis of the diaphragm and impairment of the neuromuscular innervation of breathing (60). The more rostral the level of spinal injury, the greater the risk of severe respiratory complications (61). However, traumatic injury may also lead to SCI or spinal cord oedema below the C3 to C5 spinal level, therefore affecting innervation to the thoracic and abdominal musculature which contribute to ventilation, in addition to the diaphragm (60). Furthermore, there may be a period of spinal shock; a transient state of depressed spinal reflex activity below the level of the injury. This results in flaccid paralysis of the intercostal or abdominal muscles, leading to paradoxical inward depression of the ribs during inspiration, less efficient ventilation and risk of distal airway atelectasis (61).

Additionally, expiratory muscle weakness prevents the forceful contraction necessary to evoke large pressure swings in the chest and abdomen in order generate sufficient expiratory flow to produce an effective cough (62). After SCI above thoracic spine level T12, cough effort is diminished due to expiratory muscle weakness of the diaphragm, intercostals and abdominals (63–65). Peak expiratory flow reduces below 50% of predicted values and forced vital capacity is reduced to 80–90% of predicted normal values in paraplegic and 20–60% in tetraplegic SCI patients; therefore reducing the effectiveness of cough and secretion clearance (61, 63, 66). Due to the severe consequences of respiratory muscle weakness and paralysis upon cough effort and lung volumes, respiratory secretions accumulate and contribute to increased risk of respiratory complications such as pneumonia. Respiratory disorders are the leading cause of death in patients with SCI, with half of all tetraplegic patients developing pneumonia during their initial period of hospitalisation (65). The risk of pneumonia persists long term after SCI, as chest wall compliance is further reduced by chronic stiffness of the ribcage coupled with respiratory muscle weakness (63).

Diaphragm weakness may also occur due to the secondary effects of critical illness proceeding major trauma (67). Muscle wasting occurs rapidly during critical illness, with reports of 20% muscle loss within just 1 week (68–71). ICU acquired weakness (ICUAW) is a consequence of acute illness and may include axonal polyneuropathy, myopathy and muscle atrophy, involving the diaphragm, accessory muscles and abdominal muscles as well as the phrenic nerve (72–74). ICUAW affects 25 to 100% of the critical care population with clinical risk factors including sepsis, use of neuromuscular blocking agents, steroids, deep sedation and hyperglycaemia (72, 75). The mechanisms of ICU-AW involves a complex interplay between inflammation, bioenergetic failure, disrupted metabolic homeostasis and microvascular flow, which leads to both loss of strength and atrophy of skeletal and respiratory muscles (73). Furthermore, if mechanical ventilation is necessitated, the respiratory muscles are subsequently unloaded, leading to reduced diaphragm function by 35% and diaphragm weakness occurring within 12 h of initiation (76–78). This can prolong the duration of mechanical ventilation by 2 to 7-fold, resulting in a continuous cycle of muscle weakness, ventilator dependency and risk of pneumonia (73, 77, 79, 80).

## 5 Airway protection and aspiration risks

The upper respiratory tract, including the larynx and pharynx, play a crucial role in the functions of breathing, swallowing and airway protection. Maxillofacial trauma can subsequently cause upper airway impairments in multiple ways; including blockage of the nasopharyngeal airway and oropharynx due to fractures to the maxilla, mandible or base of skull, or due to bleeding, debris (such as loose bone fragments and teeth), soft tissue swelling and oedema (81, 82). In addition to airway obstruction, aspiration of blood, vomit or oral secretions to the lower respiratory tract may occur due to restrictions in the normal protective mechanisms of coughing and swallowing, exacerbated by oedema and pain after facial injuries (81, 82). Aspiration is defined as inhalation of foreign material into the airways, leading to bacterial infiltration of the lungs and risk of pneumonia (83). The risk of aspiration of blood or oral secretions into the lower respiratory tract may also be caused by impaired motor function due to cranial nerve injury. Facial trauma may cause glossopharyngeal, hypoglossal and vagus nerve injury, each of which contribute to swallowing and gag reflexes by innervation of glottis closure, tongue movement and pharyngeal constriction (82, 84).

Disorders of consciousness, such as following traumatic brain injury (TBI), may also impair the natural motor and sensory functions of swallowing that are required to protect the airway from pulmonary aspiration (85). Swallowing is mediated by the central nervous system, including regions of the brainstem and cerebral cortex such as the sensorimotor cortex, cerebellum, cingulate gyrus, basal ganglia and the parieto-occipital lobes (86–89). Dysphagia is therefore common after TBI and is associated with oropharyngeal dysfunction, cognitive deficits and impaired consciousness, which impairs the ability to independently swallow, clear oral secretions and prevent aspiration (28, 90). Loss of consciousness and aspiration immediately after traumatic injury may also be influenced by coinciding drug or alcohol use, with intoxication occurring in approximately 10–15% of major trauma cases and in 50% all trauma-associated deaths (91, 92).

Dysphagia is also frequently occurring after spinal cord injury, with incidence rates ranging from 16 to 60% (93, 94). High cervical cord injuries may directly lead to aspiration due to impaired motor innervation of the muscles of swallowing, due to nerve damage of the ansa cervicalis, which includes fibres of C1-3 spinal nerves and the hypoglossal cranial nerve (93). Furthermore, the normal mechanics of swallowing may also be restricted by the use of cervical orthoses, such as neck braces or collars, that are frequently used to stabilise the injury site and minimise movement during the recovery period after acute injury (95, 96). In addition, the disturbances to diaphragm innervation after spinal cord injury as previously discussed, may also impair respiratory-swallowing coordination, leading to mis-timed inspiration during swallowing and potential for airway invasion (93). This aspiration risk is further exacerbated by impaired cough effort which serves as a protective reflex, due to expiratory respiratory muscle weakness after spinal cord injury (93). The motor and sensory impairment associated with ICU-AW can also involve the pharyngeal muscles; further increasing aspiration and pneumonia risk due to impaired cough and swallow (74).

Due to the vast implications of traumatic injury upon airway patency, national guidelines for major trauma management recommend rapid sequence induction of anaesthesia and intubation for any patient who cannot maintain their airway (5). Such patients therefore necessitate airway protection via endotracheal intubation and mechanical ventilation and thus face secondary risks of ventilator induced pneumonia (28, 52, 97). Furthermore, endotracheal intubation in major trauma is frequently performed emergently in pre-hospital or chaotic resuscitation settings. These suboptimal conditions drastically increase the likelihood of immediate macro-aspiration of blood or gastric contents prior to the airway being secured. In the ICU, over 40% of critically ill trauma patients who require mechanical ventilation will develop pneumonia (15, 30). Mechanical ventilation breaches the natural protective barriers of the upper airway, allowing direct communication to the tracheobronchial tree via the endotracheal tube (ETT) (98, 99). Airway intubation causes epithelial damage, dampens mucociliary clearance mechanisms and impairs the normal physiological reflexes of coughing and swallowing (98, 100, 101). These effects are exacerbated by positive pressure ventilation, immobilisation and sedative or paralytic agents, causing further respiratory muscle weakness, mucociliary dysfunction, risk of aspiration and reduced airway humidification (72, 74, 98, 100, 102–104). Epithelial cough receptors are sensitive to the chemical stimuli of sedative agents such as propofol, subsequently impairing the defensive reflex of coughing (105, 106). Furthermore, the use of general anaesthetics cause relaxation of the lower and upper oesophageal sphincter, impairing the normal physiological mechanisms that prevent gastric acid reflux and aspiration of gastric contents (105). Although necessary interventions in the event of critical illness or surgical requirement, these therapies result in an accumulation of respiratory secretions and further increase the risk of aspiration and pneumonia after major trauma (19, 107, 108).

## 6 Brain-lung interaction

**Figure 5:**
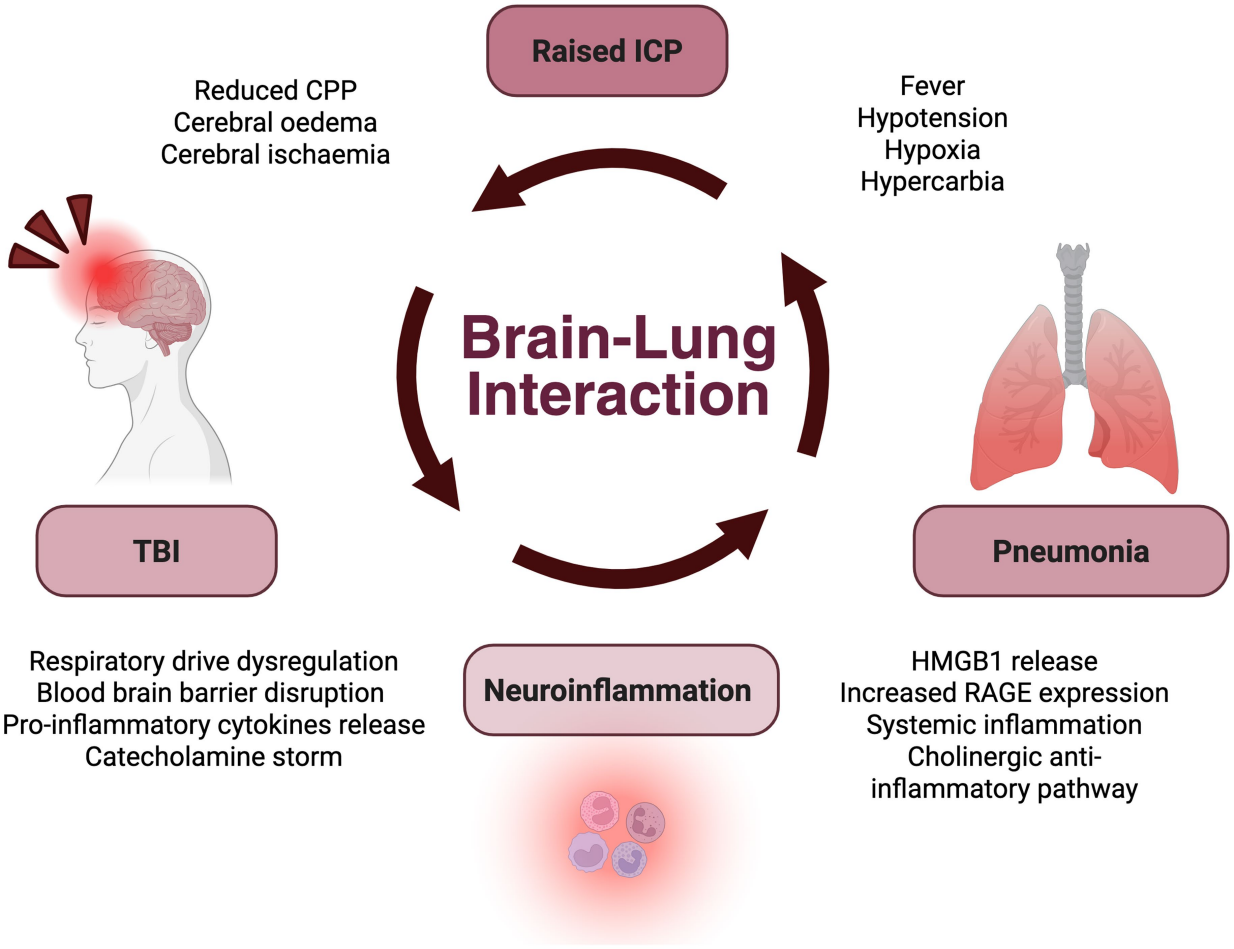
Brain to lung interaction after traumatic brain injury (TBI). Interaction between the brain and lung following TBI, contributing to a viscous cycle of neuro-inflammation and raised intracranial pressure (ICP), exacerbating the risks of pneumonia and further brain injury. Created in BioRender.

The brain and lung interaction is complex; with pathways from the brain to the lung and the lung to the brain all contributing to the “double hit” model of self-perpetuating inflammation, infective complications and secondary brain injury after TBI (37, 109) (Figure 5). Severe TBI (defined by a Glasgow Come Scale (GCS) score of 3 to 8) is an independent risk factor for the development of pneumonia (110–113). Approximately half of all TBI patients will develop pneumonia, with most of all infectious complications occurring within 3 days of acute injury (13, 28, 39, 114). Unfortunately, when pneumonia occurs after TBI it has disastrous consequences upon neurological recovery and is an independent predictor of poor outcome for up to 5 years after injury (115–117). Common clinical features of pneumonia, including fever, hypotension and hypoxia, all contribute to increased intracranial hypertension and risk of secondary brain injury (39, 118, 119). Furthermore, the impairments in respiratory function induced by pneumonia can also lead to an elevation of the partial pressures of carbon dioxide in arterial blood (PaC02), driving vasodilation of the cerebral arteries, further increasing intracranial pressure (ICP) (120). This contributes to a vicious cycle of brain injury and risk of pneumonia. Patients with severe TBI therefore frequently require mechanical ventilation and advanced respiratory support in the ICU (121). This serves to provide haemodynamic modulation through the control of carbon dioxide levels and ventilation, whilst also providing airway protection, due to loss of airway reflexes in the event of impaired consciousness (121).

TBI can cause damage to the respiratory centres of the brain, either through direct injury to the brainstem, or indirectly due to elevated ICP, cerebral haemorrhage or oedema (122). Respiratory drive can be defined as the intensity of the output of the respiratory centres of the brainstem, directly influencing neuromuscular transmission and respiratory muscle function, to ensure adequate ventilation and gas exchange (122, 123). Respiratory drive originates at the respiratory centres in the medullar and pons of the brainstem, where there are central chemoreceptors which detect chemical changes in pH, influenced by carbon dioxide levels (123). Carbon dioxide rapidly diffuses across the blood brain barriers to influence the pH of the cerebrospinal spinal fluid (CSF), directly influencing the drive to breathe. After TBI there may be significant disruption to respiratory drive, causing patients to present with dysregulated breathing patterns, including hyperventilation, periodic breathing or irregular breathing, leading to hypercapnia and respiratory compromise (122, 124). Although mechanical ventilation may be vital in such circumstances, this can lead to localised inflammation, termed biotrauma, and ventilator induced lung injury; exacerbating the secondary hit of infection risk (38, 109). Compared to non-neurological patients, individuals who require mechanical ventilation after TBI experience longer ICU stays, extended periods of mechanical ventilation and an increased risk of pneumonia and mortality (109).

In addition to ventilation-induced inflammation, TBI also leads to a complex inflammatory and immunosuppressive cascade. This combination of localised neuroinflammation and systemic inflammation exacerbates the risk of respiratory compromise and infection (28, 118, 125). Following TBI, astrocytes activate and proliferate, termed astrogliosis, leading to blood–brain-barrier disruption (126, 127). Glial cells also initiate neutrophil activation and the release of proinflammatory cytokines such as TNF-*α*, interleukin (IL)-1, IL-8 and IL-6, and reactive-oxygen species, which cross the blood–brain barrier into systemic circulation (37, 120, 126, 127). Damage to astrocytes, microglia and neurons of the blood–brain barrier also releases damage associated molecular patterns (DAMPs), which in turn can lead to endothelial and epithelial damage (28). In particular, high-mobility group-box protein 1 (HMGB1) is a DAMP that binds to receptors for advanced glycation end products (RAGE), which are highly expressed in the pulmonary endothelium and alveolar cells (28, 128). The “first hit” of the cerebral and systemic inflammation triggered by the catecholamine storm increases the susceptibility of the lungs to further inflammation, infection and secondary complications (37, 109).

The prolonged release of high-mobility group-box protein 1 (HMGB1) contributes to systemic immunosuppression and the activation of the cholinergic anti-inflammatory pathway (28). This pathway reduces inflammation via the activation of T cells in response to noradrenaline, which then release acetylcholine to inhibit inflammatory cytokines and chemokines (28). HMGB1 also has anti-inflammatory effects on macrophages, as receptors for advanced glycation end products activation downregulates toll-like receptor 4 which leads to macrophage necrosis (28). Furthermore, AMPK (Adenosine Monophosphate-activated Protein Kinase) is downregulated, which impairs phagocytosis and bacterial clearance by macrophages and neutrophils (28, 118). Collectively, these immunosuppressive features further increase the risk of infection, contributing to the “double hit” model of brain-lung crosstalk (37, 109).

Finally, TBI leads to a catecholamine surge due to the stimulation of the adrenal glands and release of noradrenaline and adrenaline in response to a rapid and abrupt increase in ICP after injury (28, 122, 129). This sympathetic storm results in vasoconstriction, increased systemic vascular resistance and increased blood pressure in the pulmonary arteries and capillaries, causing vascular permeability and capillary leakage into the alveoli of the lung (120). The increase in intravascular pressure also leads to endothelial damage and rupturing of the alveolar-capillary membranes, termed “blast theory” (120, 128). As a result, neurogenic pulmonary oedema (NPE) occurs, in which there is an accumulation of protein-rich fluid into the alveoli and pulmonary interstitial space (28, 129). The subsequent hypoxia that occurs due to impaired alveolar gas exchange is a further secondary insult associated with poor neurological recovery and patient outcomes (128).

## 7 The diagnostic conundrum of TAP

Given the complex and heterogenous mechanisms and risk factors underlying TAP, its diagnosis remains notoriously challenging. The clinical presentation of TAP often overlaps with other common conditions after traumatic injury, such as NPE, acute respiratory distress syndrome (ARDS) and sepsis, making the differentiation of TAP particularly difficult (20). Traditionally pneumonia diagnosis relies on a combination of clinical, radiographic and laboratory criteria; with guidelines in place for HAP (14, 130–134). However, many of the common clinical and radiological diagnostic tools which form the foundation of early suspicion of pneumonia and trigger microbiological investigation, present challenges in major trauma (17, 135–138) (Table 1). Traditional diagnostic scoring systems, such as the Clinical Pulmonary Infection Score (CPIS), are notoriously unreliable in major trauma cohorts. Parameters integral to these scores, such as fever, leukocytosis, and altered oxygenation, are frequently driven by the sterile systemic inflammatory response to injury rather than an isolated bacterial infection. Consequently, diagnosis of TAP lacks specificity and sensitivity, risking delayed diagnosis or over-estimated incidence rates (139, 140).

**Table 1:** Challenges of pneumonia diagnosis in major trauma patients and alternative differential diagnosis for cardinal clinical and radiological features (14, 130).

Diagnostic features of pneumonia		Potential differential diagnosis in trauma patients and diagnostic challenges
Clinical	Fever (core temperature >38.5C or <36C)	Centrally driven due to TBI. Autonomic dysreflexia in SCI. Shock or major haemorrhage. Altered thermoregulation after major burn. Artificial temperature management in ICU. Infection of any source (wound, pin sites).
	Blood leucocytosis (>10,000 leucocytes/mm^3^) or leukopenia (<4,000/mm^3^)	Response to traumatic injury itself. Surgical procedures. Infection of any source.
	Purulent tracheal secretions	Aspiration. Tracheobronchitis. Poor cough / swallow due to low GCS, sedative or opioid medication or the use of airway devices and mechanical ventilation. Smoke inhalation injury.
	Haemodynamic instability including tachycardia and hypotension	Centrally driven due to TBI. Autonomic dysreflexia in SCI. Shock or major haemorrhage. Infection of any source.
	Increased oxygen requirements	Pulmonary contusions. Blast lung injury. Acute respiratory distress syndrome. Neurogenic pulmonary oedema post TBI. Smoke inhalation injury. Post-operative atelectasis. Reduced respiratory drive due to opioids. Pain impairing ventilation, e.g., rib fractures.
Radiological	New and/or persistent changes on chest X-ray, including infiltrates	Pulmonary contusions. Blast lung injury. Acute respiratory distress syndrome. Neurogenic pulmonary oedema post TBI. Smoke inhalation injury. Post-operative atelectasis.

ICU, intensive care unit; GCS, Glasgow coma scale; SCI, spinal cord injury; TBI, traumatic brain injury.

Although preventative antimicrobial therapy may offer early, protective cover for TAP, the rapidly rising prevalence of multi-drug resistant nosocomial infections means the management of pneumonia is becoming increasingly challenging, particularly in complex and heterogenous cohorts such as those with major trauma (15, 141). In the trauma ICU, multi-drug resistant pathogens occur in approximately a third of all patients; including polymicrobial organisms, methicillin-resistant *Staphylococcus aureus* (MRSA) and *Escherichia coli* extended spectrum β -lactamase (ESBL) (142, 143). Traditional culture methods, such as sputum samples, are often slow (48–72 h), have low sensitivity for co-pathogens, are often dominated by faster growing organisms, with risk of contamination (144). Delayed or misdiagnosis may risk poor outcomes due to belated treatment.

### 7.1 Future directions—artificial intelligence

Artificial intelligence (AI) is a rapidly growing tool which has demonstrated transformative potential across multiple sectors of modern healthcare, offering innovative and sophisticated diagnostic solutions to complex clinical practise, such as that faced by major trauma (52, 145). AI models have the potential to synthesise complex data from electronic health records to predict which patients are at higher risk for pneumonia (146–149). Previous studies have demonstrated the potential benefit of AI for early detection of VAP in critical care patients (150, 151). Furthermore, AI algorithms may also analyse lab results and imaging to aid diagnostic consistency and efficiency; as demonstrated in previous studies using AI for imaging interpretation to support the diagnosis of TBI and COVID-19 induced pneumonia (150, 152–154). Specific to TAP, recent studies have utilised machine learning and predictive models for pneumonia in patients with TBI (155–158), blast-injured combat trauma (159) and flail chest (49); using a combination of clinically-available data, laboratory results and imaging. Given the heterogeneity of major trauma, future AI applications should prioritise multimodal platforms that integrate continuous physiological monitoring, electronic health record data, and advanced imaging, to derive highly personalised predictive TAP phenotypes. In parallel, emerging genomic approaches, including rapid molecular and metagenomic diagnostics, could be incorporated alongside these datasets. Such integration has the potential to enhance pathogen identification through earlier, culture-independent detection and antimicrobial resistance profiling. Collectively, these advances may support precision medicine by enabling targeted therapy, thereby reducing treatment delays, unnecessary antibiotic use, and mitigating the development of multidrug resistance. However, the generalizability of such models requires external validation prior to bedside implementation (156, 159). Although AI offers huge potential to support diagnostic challenges and practitioner uncertainty in TAP, further research is necessary to improve the validity, reliability and trustworthiness and to ensure patient safety.

## 8 Conclusion

The pathophysiology of TAP is complex, with a breath of potential underlying risk factors and mechanisms arising from heterogenous traumatic injuries, clinical management strategies and immune system remodelling. Consequently, the diagnosis of pneumonia after traumatic injury is challenging, complicated by numerous overlapping clinical conditions and a vast array of potential microbiological sources. Current diagnostic guidelines for HAP fail to address the complexities and intricacies inherent to the major trauma cohort. Further work is urgently required to enhance the understanding of TAP, with further recognition as its own unique pathology. Underpinning the specific immune dysregulation and inflammatory response to trauma-associated pneumonia, in addition to injury itself, may aid the development of specific biomarkers and novel therapeutic targets. Furthermore, artificial intelligence and machine-learning based stratification may also assist clinicians with the interpretation of complex clinical information and enable precise, early detection. Future research should prioritise the development of standardised diagnostic protocols tailored to TAP. Ultimately, with improved understanding and evidence-based guidelines specific to TAP, clinical management can be optimised to improve both short- and long-term outcomes for patients following major trauma.

## References

[1] Thompson L Hill M Lecky F Shaw G. Defining major trauma: a Delphi study. Scand J Trauma Resusc Emerg Med. (2021) 29:63. doi: 10.1186/s13049-021-00870-w, 33971922 PMC8108467

[2] Thompson L Hill M Shaw G. Defining major trauma: a literature review. Br Paramed J. (2019) 4:22–30. doi: 10.29045/14784726.2019.06.4.1.22, 33328825 PMC7706773

[3] Moran CG Lecky F Bouamra O Lawrence T Edwards A Woodford M . Changing the system - major trauma patients and their outcomes in the NHS (England) 2008–17. EClinicalMedicine. (2018) 2:13–21. doi: 10.1016/j.eclinm.2018.07.001 31193723 PMC6537569

[4] Dogrul BN Kiliccalan I Asci ES Peker SC. Blunt trauma related chest wall and pulmonary injuries: an overview. Chin J Traumatol. (2020) 23:125–38. doi: 10.1016/j.cjtee.2020.04.003, 32417043 PMC7296362

[5] National Institute for Health and Care Excellence. Major Trauma: Assessment and Initial Management. National Guideline NG39. (2016). Available online at: https://www.nice.org.uk/guidance/ng39/resources/major-trauma-assessment-and-initial-management-pdf-1837400761285 (Accessed September 1, 2025).

[6] Maddock A Corfield AR Donald MJ Lyon RM Sinclair N Fitzpatrick D . Prehospital critical care is associated with increased survival in adult trauma patients in Scotland. Emerg Med J. (2020) 37:141–5. doi: 10.1136/emermed-2019-208458, 31959616 PMC7057794

[7] Tien H Chu PTY Brenneman F. Causes of death following multiple trauma. Curr Orthop. (2004) 18:304–10. doi: 10.1016/j.cuor.2004.04.006

[8] Trunkey D. Accidental and intentional injuries account for more years of life lost in the US than cancer and heart disease. Among the prescribed remedies are improved preventive efforts, speedier surgery and further research. Sci Am. (1983) 249:28–35. doi: 10.1038/scientificamerican0883-28 6623052

[9] Sobrino J Shafi S. Timing and causes of death after injuries. Proc (Bayl Univ Med Cent). (2013) 26:120–3. doi: 10.1080/08998280.2013.11928934, 23543966 PMC3603725

[10] Femling JK West SD Hauswald EK Gresham HD Hall PR. Nosocomial infections after severe trauma are associated with lower apolipoproteins B and AII. J Trauma Acute Care Surg. (2013) 74:1067–73. doi: 10.1097/TA.0b013e3182826be0, 23511146 PMC3914009

[11] Coccolini F Rausa E Montori G Fugazzola P Ceresoli M Sartelli M . Risk factors for infections in trauma patients. Curr Trauma Rep. (2017) 3:285–91. doi: 10.1007/s40719-017-0094-y

[12] National Institute for Health and Care Excellence. Pneumonia (Hospital-Acquired): Antimicrobial Prescribing. National Institute for Health and Care Excellence; (2019). Available online at: https://www.nice.org.uk/guidance/ng139/resources (Accessed September 1, 2025).

[13] Howroyd F Sardeli AV Smith FG Veenith T Duggal NA Ahmed Z. Biomarkers for pneumonia after major trauma: a systematic review and meta-analysis. J Intensive Care Soc. (2025) 27:17511437251344068. doi: 10.1177/17511437251344068, 40520925 PMC12165960

[14] American Thoracic Society; Infectious Diseases Society of America. Guidelines for the management of adults with hospital-acquired, ventilator-associated, and healthcare-associated pneumonia. Am J Respir Crit Care Med. (2005) 171:388–416. doi: 10.1164/rccm.200405-644ST 15699079

[15] Schellenberg M Inaba K. Pneumonia in Trauma Patients. Curr Trauma Rep. (2017) 3:308–14. doi: 10.1007/s40719-017-0105-z, 32226724 PMC7100823

[16] Glance LG Stone PW Mukamel DB Dick AW. Increases in mortality, length of stay, and cost associated with hospital-acquired infections in trauma patients. Arch Surg. (2011) 146:794–801. doi: 10.1001/archsurg.2011.41, 21422331 PMC3336161

[17] Mangram AJ Sohn J Zhou N Hollingworth AK Ali-Osman FR Sucher JF . Trauma-associated pneumonia: time to redefine ventilator-associated pneumonia in trauma patients. Am J Surg. (2015) 210:1056–61. doi: 10.1016/j.amjsurg.2015.06.029 26477792

[18] Lord JM Midwinter MJ Chen Y-F Belli A Brohi K Kovacs EJ . The systemic immune response to trauma: an overview of pathophysiology and treatment. Lancet. (2014) 384:1455–65. doi: 10.1016/S0140-6736(14)60687-5, 25390327 PMC4729362

[19] Mackenzie G. The definition and classification of pneumonia. Pneumonia. (2016) 8:14. doi: 10.1186/s41479-016-0012-z, 28702293 PMC5471962

[20] Patel CB Gillespie TL Goslar PW Sindhwani M Petersen SR. Trauma-associated pneumonia in adult ventilated patients. Am J Surg. (2011) 202:66–70. doi: 10.1016/j.amjsurg.2010.10.010, 21497790

[21] Hazeldine J Lord JM Belli A. Traumatic brain injury and peripheral immune suppression: primer and prospectus. Front Neurol. (2015) 6:235. doi: 10.3389/fneur.2015.00235, 26594196 PMC4633482

[22] Osuka A Ogura H Ueyama M Shimazu T Lederer JA. Immune response to traumatic injury: harmony and discordance of immune system homeostasis. Acute Med Surg. (2014) 1:63–9. doi: 10.1002/ams2.17, 29930824 PMC5997205

[23] Asehnoune K Roquilly A Abraham E. Innate immune dysfunction in trauma patients: from pathophysiology to treatment. Anesthesiology. (2012) 117:411–6. doi: 10.1097/ALN.0b013e31825f018d, 22728780

[24] Stoecklein VM Osuka A Lederer JA. Trauma equals danger--damage control by the immune system. J Leukoc Biol. (2012) 92:539–51. doi: 10.1189/jlb.0212072, 22654121 PMC3427603

[25] Tullie S Nicholson T Bishop JRB McGee KC Asiri A Sullivan J . Severe thermal and major traumatic injury results in elevated plasma concentrations of total heme that are associated with poor clinical outcomes and systemic immune suppression. Front Immunol. (2024) 15:1416820. doi: 10.3389/fimmu.2024.1416820, 38947312 PMC11211257

[26] Horner E Lord JM Hazeldine J. The immune suppressive properties of damage associated molecular patterns in the setting of sterile traumatic injury. Front Immunol. (2023) 14:1239683. doi: 10.3389/fimmu.2023.1239683 37662933 PMC10469493

[27] Manson J Thiemermann C Brohi K. Trauma alarmins as activators of damage-induced inflammation. Br J Surg. (2012) 99:12–20. doi: 10.1002/bjs.7717 22441851

[28] Hu PJ Pittet J-F Kerby JD Bosarge PL Wagener BM. Acute brain trauma, lung injury, and pneumonia: more than just altered mental status and decreased airway protection. Am J Physiol Lung Cell Mol Physiol. (2017) 313:L1–L15. doi: 10.1152/ajplung.00485.2016, 28408366

[29] Hazeldine J Hampson P Lord JM. The impact of trauma on neutrophil function. Injury. (2014) 45:1824–33. doi: 10.1016/j.injury.2014.06.021, 25106876

[30] Leonard JM Zhang CX Lu L Hoofnagle MH Fuchs A Clemens RA . Extrathoracic multiple trauma dysregulates neutrophil function and exacerbates pneumonia-induced lung injury. J Trauma Acute Care Surg. (2021) 90:924–34. doi: 10.1097/TA.0000000000003147, 34016916 PMC8932930

[31] Papayannopoulos V. Neutrophil extracellular traps in immunity and disease. Nat Rev Immunol. (2018) 18:134–47. doi: 10.1038/nri.2017.105, 28990587

[32] Timmermans K Kox M Vaneker M van den Berg M John A van Laarhoven A . Plasma levels of danger-associated molecular patterns are associated with immune suppression in trauma patients. Intensive Care Med. (2016) 42:551–61. doi: 10.1007/s00134-015-4205-3, 26912315 PMC5413532

[33] Gentile LF Cuenca AG Efron PA Ang D Bihorac A McKinley BA . Persistent inflammation and immunosuppression: a common syndrome and new horizon for surgical intensive care. J Trauma Acute Care Surg. (2012) 72:1491–501. doi: 10.1097/TA.0b013e318256e000 22695412 PMC3705923

[34] Ward NS Casserly B Ayala A. The compensatory anti-inflammatory response syndrome (CARS) in critically ill patients. Clin Chest Med. (2008) 29:617–25, viii. doi: 10.1016/j.ccm.2008.06.010, 18954697 PMC2786900

[35] Thompson KB Krispinsky LT Stark RJ. Late immune consequences of combat trauma: a review of trauma-related immune dysfunction and potential therapies. Mil Med Res. (2019) 6:11. doi: 10.1186/s40779-019-0202-0, 31014397 PMC6480837

[36] Mira JC Brakenridge SC Moldawer LL Moore FA. Persistent inflammation, immunosuppression and catabolism syndrome. Crit Care Clin. (2017) 33:245–58. doi: 10.1016/j.ccc.2016.12.001, 28284293 PMC5351769

[37] Ziaka M Exadaktylos A. Brain–lung interactions and mechanical ventilation in patients with isolated brain injury. Crit Care. (2021) 25:358. doi: 10.1186/s13054-021-03778-0, 34645485 PMC8512596

[38] Ziaka M Exadaktylos A. ARDS associated acute brain injury: from the lung to the brain. Eur J Med Res. (2022) 27:150. doi: 10.1186/s40001-022-00780-2, 35964069 PMC9375183

[39] Lim HB Smith M. Systemic complications after head injury: a clinical review. Anaesthesia. (2007) 62:474–82. doi: 10.1111/j.1365-2044.2007.04998.x, 17448059

[40] Chapman BC Herbert B Rodil M Salotto J Stovall RT Biffl W . Ribscore: a novel radiographic score based on fracture pattern that predicts pneumonia, respiratory failure, and tracheostomy. J Trauma Acute Care Surg. (2016) 80:95–101. doi: 10.1097/TA.0000000000000867 26683395

[41] Brasel KJ Guse CE Layde P Weigelt JA. Rib fractures: relationship with pneumonia and mortality. Crit Care Med. (2006) 34:1642–6. doi: 10.1097/01.CCM.0000217926.40975.4B, 16625122

[42] Kim M Moore JE. Chest trauma: current recommendations for rib fractures, pneumothorax, and other injuries. Curr Anesthesiol Rep. (2020) 10:61–8. doi: 10.1007/s40140-020-00374-w, 32435162 PMC7223697

[43] Karmakar MK Ho AM. Acute pain management of patients with multiple fractured ribs. J Trauma. (2003) 54:615–25. doi: 10.1097/01.TA.0000053197.40145.62, 12634549

[44] Marco CA Sorensen D Hardman C Bowers B Holmes J McCarthy MC. Risk factors for pneumonia following rib fractures. Am J Emerg Med. (2020) 38:610–2. doi: 10.1016/j.ajem.2019.10.021, 31831351

[45] Tiwari A Nair S Baker A. "The pathophysiology of flail chest injury". In: McKee MD Schemitsch EH, editors. Injuries to the Chest Wall: Diagnosis and Management. Cham: Springer International Publishing (2015). p. 19–32.

[46] Ehrnthaller C Flierl M Perl M Denk S Unnewehr H Ward PA . The molecular fingerprint of lung inflammation after blunt chest trauma. Eur J Med Res. (2015) 20:70. doi: 10.1186/s40001-015-0164-y, 26303896 PMC4548898

[47] Dolgachev VA Yu B Reinke JM Raghavendran K Hemmila MR. Host susceptibility to gram-negative pneumonia after lung contusion. J Trauma Acute Care Surg. (2012) 72:614–22. doi: 10.1097/TA.0b013e318243d9b1, 22491544 PMC3576878

[48] Battle CE Evans PA. Predictors of mortality in patients with flail chest: a systematic review. Emerg Med J. (2015) 32:961–5. doi: 10.1136/emermed-2015-204939, 26188067

[49] Song X Li H Chen Q Zhang T Huang G Zou L . Predicting pneumonia during hospitalization in flail chest patients using machine learning approaches. Front Surg. (2022) 9:1060691. doi: 10.3389/fsurg.2022.1060691, 36684357 PMC9852626

[50] Lee C Porter K. The prehospital management of pelvic fractures. Emerg Med J. (2007) 24:130–3. doi: 10.1136/emj.2006.041384, 17251627 PMC2658194

[51] Marco RA Stuckey RM Holloway SP. Prolonged bed rest as adjuvant therapy after complex reconstructive spine surgery. Clin Orthop Relat Res. (2012) 470:1658–67. doi: 10.1007/s11999-012-2318-3, 22467418 PMC3348306

[52] Howroyd F Chacko C MacDuff A Gautam N Pouchet B Tunnicliffe B . Ventilator-associated pneumonia: pathobiological heterogeneity and diagnostic challenges. Nat Commun. (2024) 15:6447. doi: 10.1038/s41467-024-50805-z, 39085269 PMC11291905

[53] Hess DR. Patient positioning and ventilator-associated pneumonia. Respir Care. (2005) 50:892–9. doi: 10.4187/respcare.05500892, 15972110

[54] Blot SI Poelaert J Kollef M. How to avoid microaspiration? A key element for the prevention of ventilator-associated pneumonia in intubated ICU patients. BMC Infect Dis. (2014) 14:119. doi: 10.1186/1471-2334-14-119, 25430629 PMC4289393

[55] Drakulovic MB Torres A Bauer TT Nicolas JM Nogué S Ferrer M. Supine body position as a risk factor for nosocomial pneumonia in mechanically ventilated patients: a randomised trial. Lancet. (1999) 354:1851–8. doi: 10.1016/S0140-6736(98)12251-1, 10584721

[56] Wu D Geng X Wu H Liu X Liu X Ma L . Effect of early mobilization on the development of pneumonia in patients with traumatic brain injury in the neurosurgical intensive care unit: a historical controls study. Nurs Crit Care. (2024) 29:962–73. doi: 10.1111/nicc.13067, 38639246

[57] Hussain A Hunt I. Acute diaphragmatic injuries associated with traumatic rib fractures: experiences of a major trauma Centre and the importance of intra-pleural assessment. J Chest Surg. (2021) 54:59–64. doi: 10.5090/kjtcs.20.126, 33767010 PMC7946519

[58] Karhof S Simmermacher RKJ Gerbranda P van Wessem KJP Leenen LPH Hietbrink F. Diaphragm injuries in a mature trauma system: still a diagnostic challenge. Front Surg. (2024) 11:1489260. doi: 10.3389/fsurg.2024.1489260, 39717351 PMC11663924

[59] Hwang S-W Kim H-Y Byun JH. Management of patients with traumatic rupture of the diaphragm. Korean J Thorac Cardiovasc Surg. (2011) 44:348–54. doi: 10.5090/kjtcs.2011.44.5.348, 22263186 PMC3249338

[60] Zimmer MB Kwaku N Goshgarian HG. Effect of spinal cord injury on the respiratory system: basic research and current clinical treatment options. J Spinal Cord Med. (2007) 30:319–30. doi: 10.1080/10790268.2007.11753947, 17853653 PMC2031930

[61] Berlowitz DJ Wadsworth B Ross J. Respiratory problems and management in people with spinal cord injury. Breathe (Sheff). (2016) 12:328–40. doi: 10.1183/20734735.012616, 28270863 PMC5335574

[62] Smith JA Aliverti A Quaranta M McGuinness K Kelsall A Earis J . Chest wall dynamics during voluntary and induced cough in healthy volunteers. J Physiol. (2012) 590:563–74. doi: 10.1113/jphysiol.2011.213157, 22144580 PMC3379701

[63] Baydur A Adkins RH Milic-Emili J. Lung mechanics in individuals with spinal cord injury: effects of injury level and posture. J Appl Physiol. (2001) 90:405–11. doi: 10.1152/jappl.2001.90.2.405, 11160035

[64] Wang AY Jaeger RJ Yarkony GM Turba RM. Cough in spinal cord injured patients: the relationship between motor level and peak expiratory flow. Spinal Cord. (1997) 35:299–302. doi: 10.1038/sj.sc.3100370, 9160454

[65] Burns SP. Acute respiratory infections in persons with spinal cord injury. Phys Med Rehabil Clin N Am. (2007) 18:203–216, v–vi. doi: 10.1016/j.pmr.2007.02.001, 17543769 PMC7172350

[66] Linn WS Adkins RH Gong H Waters RL. Pulmonary function in chronic spinal cord injury: a cross-sectional survey of 222 Southern California adult outpatients. Arch Phys Med Rehabil. (2000) 81:757–63. doi: 10.1016/S0003-9993(00)90107-2, 10857520

[67] Levine S Nguyen T Taylor N Friscia ME Budak MT Rothenberg P . Rapid disuse atrophy of diaphragm fibers in mechanically ventilated humans. N Engl J Med. (2008) 358:1327–35. doi: 10.1056/nejmoa070447, 18367735

[68] Dirks ML Wall BT van de Valk B Holloway TM Holloway GP Chabowski A . One week of bed rest leads to substantial muscle atrophy and induces whole-body insulin resistance in the absence of skeletal muscle lipid accumulation. Diabetes. (2016) 65:2862–75. doi: 10.2337/db15-1661, 27358494

[69] Batt J dos Santos CC Cameron JI Herridge MS. Intensive care unit-acquired weakness: clinical phenotypes and molecular mechanisms. Am J Respir Crit Care Med. (2013) 187:238–46. doi: 10.1164/rccm.201205-0954SO 23204256

[70] Puthucheary ZA Rawal J McPhail M Connolly B Ratnayake G Chan P . Acute skeletal muscle wasting in critical illness. JAMA. (2013) 310:1591–600. doi: 10.1001/jama.2013.278481, 24108501

[71] Stevens RD Marshall SA Cornblath DR Hoke A Needham DM de Jonghe B . A framework for diagnosing and classifying intensive care unit-acquired weakness. Crit Care Med. (2009) 37:S299–308. doi: 10.1097/CCM.0b013e3181b6ef67, 20046114

[72] Hermans G Van den Berghe G. Clinical review: intensive care unit acquired weakness. Crit Care. (2015) 19:274. doi: 10.1186/s13054-015-0993-7, 26242743 PMC4526175

[73] Garnacho-Montero J Amaya-Villar R García-Garmendía JL Madrazo-Osuna J Ortiz-Leyba C. Effect of critical illness polyneuropathy on the withdrawal from mechanical ventilation and the length of stay in septic patients. Crit Care Med. (2005) 33:349–54. doi: 10.1097/01.CCM.0000153521.41848.7E, 15699838

[74] Thille AW Boissier F Muller M Levrat A Bourdin G Rosselli S . Role of ICU-acquired weakness on extubation outcome among patients at high risk of reintubation. Crit Care. (2020) 24:86. doi: 10.1186/s13054-020-2807-9, 32164739 PMC7069045

[75] De Jonghe B Bastuji-Garin S Durand MC Malissin I Rodrigues P Cerf C . Respiratory weakness is associated with limb weakness and delayed weaning in critical illness. Crit Care Med. (2007) 35:17855814:2007–15. doi: 10.1097/01.ccm.0000281450.01881.d8 17855814

[76] Supinski GS Morris PE Dhar S Callahan LA. Diaphragm dysfunction in critical illness. Chest. (2018) 153:1040–51. doi: 10.1016/j.chest.2017.08.1157, 28887062 PMC6026291

[77] Petrof BJ Hussain SN. Ventilator-induced diaphragmatic dysfunction: what have we learned? Curr Opin Crit Care. (2016) 22:67–72. doi: 10.1097/MCC.0000000000000272, 26627540

[78] Powers SK Kavazis AN Levine S. Prolonged mechanical ventilation alters diaphragmatic structure and function. Crit Care Med. (2009) 37:S347–53. doi: 10.1097/CCM.0b013e3181b6e760, 20046120 PMC2909674

[79] Dres M Goligher EC Heunks LMA Brochard LJ. Critical illness-associated diaphragm weakness. Intensive Care Med. (2017) 43:1441–52. doi: 10.1007/s00134-017-4928-4, 28917004

[80] Polkey MI Walters N. Weaning strategies in problem patients. J Intensive Care Soc. (2008) 9:173–7. doi: 10.1177/175114370800900217

[81] Barak M Bahouth H Leiser Y Abu El-Naaj I. Airway management of the patient with maxillofacial trauma: review of the literature and suggested clinical approach. Biomed Res Int. (2015) 2015:724032. doi: 10.1155/2015/724032, 26161411 PMC4486512

[82] Hutchison I Lawlor M Skinner D. Abc of major trauma. Major maxillofacial injuries. BMJ. (1990) 301:595–9. doi: 10.1136/bmj.301.6752.595, 2242459 PMC1663741

[83] Raghavendran K Nemzek J Napolitano LM Knight PR. Aspiration-induced lung injury. Crit Care Med. (2011) 39:818–26. doi: 10.1097/CCM.0b013e31820a856b, 21263315 PMC3102154

[84] Esonu O Sardesai MG. Initial assessment of the facial trauma patient. Semin Plast Surg. (2021) 35:225–8. doi: 10.1055/s-0041-1735817 34819803 PMC8604615

[85] Orso D Vetrugno L Federici N D’Andrea N Bove T. Endotracheal intubation to reduce aspiration events in acutely comatose patients: a systematic review. Scand J Trauma Resusc Emerg Med. (2020) 28:116. doi: 10.1186/s13049-020-00814-w, 33303004 PMC7726605

[86] Cheng I Takahashi K Miller A Hamdy S. Cerebral control of swallowing: an update on neurobehavioral evidence. J Neurol Sci. (2022) 442:120434. doi: 10.1016/j.jns.2022.120434, 36170765

[87] Dehaghani SE Yadegari F Asgari A Chitsaz A Karami M. Brain regions involved in swallowing: evidence from stroke patients in a cross-sectional study. J Res Med Sci. (2016) 21:45. doi: 10.4103/1735-1995.183997, 27904591 PMC5122214

[88] Sasegbon A Hamdy S. The role of the cerebellum in swallowing. Dysphagia. (2023) 38:497–509. doi: 10.1007/s00455-021-10271-x, 33675425 PMC10006062

[89] Qin Y Tang Y Liu X Qiu S. Neural basis of dysphagia in stroke: a systematic review and meta-analysis. Front Hum Neurosci. (2023) 17:1077234. doi: 10.3389/fnhum.2023.1077234 36742358 PMC9896523

[90] Lee WK Yeom J Lee WH Seo HG Oh BM Han TR. Characteristics of dysphagia in severe traumatic brain injury patients: a comparison with stroke patients. Ann Rehabil Med. (2016) 40:432–9. doi: 10.5535/arm.2016.40.3.432, 27446779 PMC4951361

[91] Woliansky J Gumm K Clark N Knott J Read DJ. Drug and alcohol intoxication in major trauma: associations, trends and outcomes over a decade. Emerg Med Australas. (2023) 35:792–8. doi: 10.1111/1742-6723.14231, 37156569

[92] Hadfield RJ Mercer M Parr MJ. Alcohol and drug abuse in trauma. Resuscitation. (2001) 48:25–36. doi: 10.1016/s0300-9572(00)00315-4, 11162880

[93] McRae J Morgan S Wallace E Miles A. Oropharyngeal dysphagia in acute cervical spinal cord injury: a literature review. Dysphagia. (2023) 38:1025–38. doi: 10.1007/s00455-022-10535-0, 36374337 PMC10326135

[94] Chaw E Shem K Castillo K Wong SL Chang J. Dysphagia and associated respiratory considerations in cervical spinal cord injury. Top Spinal Cord Inj Rehabil. (2012) 18:291–9. doi: 10.1310/sci1804-291, 23459678 PMC3584789

[95] Morishima N Ohota K Miura Y. The influences of halo-vest fixation and cervical hyperextension on swallowing in healthy volunteers. Spine. (2005) 30:E179–82. doi: 10.1097/01.brs.0000157475.47514.75, 15803067

[96] Stambolis V Brady S Klos D Wesling M Fatianov T Hildner C. The effects of cervical bracing upon swallowing in young, normal, healthy volunteers. Dysphagia. (2003) 18:39–45. doi: 10.1007/s00455-002-0083-2, 12497195

[97] Wu D Wu C Zhang S Zhong Y. Risk factors of ventilator-associated pneumonia in critically III patients. Front Pharmacol. (2019) 10:482. doi: 10.3389/fphar.2019.00482, 31143118 PMC6521332

[98] Mietto C Pinciroli R Patel N Berra L. Ventilator associated pneumonia: evolving definitions and preventive strategies. Respir Care. (2013) 58:990–1007. doi: 10.4187/respcare.02380, 23709196

[99] Hunter JD. Ventilator associated pneumonia. BMJ. (2012) 344:e3325. doi: 10.1136/bmj.e3325, 22645207

[100] Goetz RL Vijaykumar K Solomon GM. Mucus clearance strategies in mechanically ventilated patients. Front Physiol. (2022) 13:834716. doi: 10.3389/fphys.2022.834716, 35399263 PMC8984116

[101] Konrad F Schreiber T Brecht-Kraus D Georgieff M. Mucociliary transport in ICU patients. Chest. (1994) 105:237–41. doi: 10.1378/chest.105.1.237, 8275739

[102] Chastre J Fagon JY. Ventilator-associated pneumonia. Am J Respir Crit Care Med. (2002) 165:867–903. doi: 10.1164/ajrccm.165.7.2105078, 11934711

[103] Lorente L Lecuona M Jiménez A Mora ML Sierra A. Ventilator-associated pneumonia using a heated humidifier or a heat and moisture exchanger: a randomized controlled trial [ISRCTN88724583]. Crit Care. (2006) 10:R116. doi: 10.1186/cc5009, 16884530 PMC1750976

[104] Rouzé A Jaillette E Nseir S. Relationship between microaspiration of gastric contents and ventilator-associated pneumonia. Ann Transl Med. (2018) 6:428. doi: 10.21037/atm.2018.07.36, 30581836 PMC6275405

[105] Robinson M Davidson A. Aspiration under anaesthesia: risk assessment and decision-making. Contin Educ Anaesth Crit Care Pain. (2013) 14:171–5. doi: 10.1093/bjaceaccp/mkt053

[106] Yin N Xia J Cao YZ Lu X Yuan J Xie J. Effect of propofol combined with opioids on cough reflex suppression in gastroscopy: study protocol for a double-blind randomized controlled trial. BMJ Open. (2017) 7:e014881. doi: 10.1136/bmjopen-2016-014881, 28864688 PMC5589021

[107] Lynch JP III. Hospital-acquired pneumonia: risk factors, microbiology, and treatment. Chest. (2001) 119:373S–84S. doi: 10.1378/chest.119.2_suppl.373s 11171773

[108] Cillóniz C Torres A Niederman MS. Management of pneumonia in critically ill patients. BMJ. (2021) 375:e065871. doi: 10.1136/bmj-2021-065871, 34872910

[109] Mrozek S Constantin JM Geeraerts T. Brain-lung crosstalk: implications for neurocritical care patients. World J Crit Care Med. (2015) 4:163–78. doi: 10.5492/wjccm.v4.i3.163, 26261769 PMC4524814

[110] Hyllienmark P BrattstrÖM O Larsson E Martling CR Petersson J Oldner A. High incidence of post-injury pneumonia in intensive care-treated trauma patients. Acta Anaesthesiol Scand. (2013) 57:848–54. doi: 10.1111/aas.12111, 23550742

[111] Teasdale G Jennett B. Assessment of coma and impaired consciousness: a practical scale. Lancet. (1974) 304:81–4. doi: 10.1016/s0140-6736(74)91639-0 4136544

[112] Basak D Chatterjee S Attergrim J Sharma MR Soni KD Verma S . Glasgow coma scale compared to other trauma scores in discriminating in-hospital mortality of traumatic brain injury patients admitted to urban Indian hospitals: a multicentre prospective cohort study. Injury. (2023) 54:93–9. doi: 10.1016/j.injury.2022.09.035, 36243583

[113] Cavalcanti M Ferrer M Ferrer R Morforte R Garnacho A Torres A. Risk and prognostic factors of ventilator-associated pneumonia in trauma patients. Crit Care Med. (2006) 34:1067–72. doi: 10.1097/01.ccm.0000206471.44161.a0, 16484918

[114] Chen S Gao G Xia Y Wu Z. Incidence rate and risk factors of ventilator-associated pneumonia in patients with traumatic brain injury: a systematic review and meta-analysis of observational studies. J Thorac Dis. (2023) 15:2068–78. doi: 10.21037/jtd-23-425, 37197499 PMC10183555

[115] Kumar RG Kesinger MR Juengst SB Brooks MM Fabio A Dams-O'Connor K . Effects of hospital-acquired pneumonia on long-term recovery and hospital resource utilization following moderate to severe traumatic brain injury. J Trauma Acute Care Surg. (2020) 88:491–500. doi: 10.1097/TA.0000000000002562, 31804412 PMC7802881

[116] Kesinger MR Kumar RG Wagner AK Puyana JC Peitzman AP Billiar TR . Hospital-acquired pneumonia is an independent predictor of poor global outcome in severe traumatic brain injury up to 5 years after discharge. J Trauma Acute Care Surg. (2015) 78:396–402. doi: 10.1097/TA.0000000000000526, 25757128 PMC5070940

[117] Prieto-Alvarado DE Parada-Gereda HM Molano D Martinez YL Tafurt GPR Masclans J-R. Risk factors and outcomes of ventilator-associated pneumonia in patients with traumatic brain injury: a systematic review and meta-analysis. J Crit Care. (2025) 85:154922. doi: 10.1016/j.jcrc.2024.154922, 39362181

[118] Sharma R Shultz SR Robinson MJ Belli A Hibbs ML O'Brien TJ . Infections after a traumatic brain injury: the complex interplay between the immune and neurological systems. Brain Behav Immun. (2019) 79:63–74. doi: 10.1016/j.bbi.2019.04.034, 31029794

[119] Bronchard R Albaladejo P Brezac G Geffroy A Seince PF Morris W . Early onset pneumonia: risk factors and consequences in head trauma patients. Anesthesiology. (2004) 100:234–9. doi: 10.1097/00000542-200402000-00009 14739794

[120] Chacón-Aponte AA Durán-Vargas ÉA Arévalo-Carrillo JA Lozada-Martínez ID Bolaño-Romero MP Moscote-Salazar LR . Brain-lung interaction: a vicious cycle in traumatic brain injury. Acute Crit Care. (2022) 37:35–44. doi: 10.4266/acc.2021.01193, 35172526 PMC8918716

[121] Robba C Poole D McNett M Asehnoune K Bösel J Bruder N . Mechanical ventilation in patients with acute brain injury: recommendations of the European Society of Intensive Care Medicine consensus. Intensive Care Med. (2020) 46:2397–410. doi: 10.1007/s00134-020-06283-0, 33175276 PMC7655906

[122] Frisvold S Coppola S Ehrmann S Chiumello D Guérin C. Respiratory challenges and ventilatory management in different types of acute brain-injured patients. Crit Care. (2023) 27:247. doi: 10.1186/s13054-023-04532-4, 37353832 PMC10290317

[123] Jonkman AH de Vries HJ Heunks LMA. Physiology of the respiratory drive in ICU patients: implications for diagnosis and treatment. Crit Care. (2020) 24:104. doi: 10.1186/s13054-020-2776-z, 32204710 PMC7092542

[124] North JB Jennett S. Abnormal breathing patterns associated with acute brain damage. Arch Neurol. (1974) 31:338–44. doi: 10.1001/archneur.1974.00490410086010, 4411797

[125] Meisel C Schwab JM Prass K Meisel A Dirnagl U. Central nervous system injury-induced immune deficiency syndrome. Nat Rev Neurosci. (2005) 6:775–86. doi: 10.1038/nrn1765, 16163382

[126] Burda JE Bernstein AM Sofroniew MV. Astrocyte roles in traumatic brain injury. Exp Neurol. (2016) 275:305–15. doi: 10.1016/j.expneurol.2015.03.020 25828533 PMC4586307

[127] He L Zhang R Yang M Lu M. The role of astrocyte in neuroinflammation in traumatic brain injury. Biochim Biophys Acta. (2024) 1870:166992. doi: 10.1016/j.bbadis.2023.166992, 38128844

[128] Koutsoukou A Katsiari M Orfanos SE Kotanidou A Daganou M Kyriakopoulou M . Respiratory mechanics in brain injury: a review. World J Crit Care Med. (2016) 5:65–73. doi: 10.5492/wjccm.v5.i1.65, 26855895 PMC4733457

[129] Davison DL Terek M Chawla LS. Neurogenic pulmonary edema. Crit Care. (2012) 16:212. doi: 10.1186/cc11226, 22429697 PMC3681357

[130] Masterton RG Galloway A French G Street M Armstrong J Brown E . Guidelines for the management of hospital-acquired pneumonia in the UK: report of the working party on hospital-acquired pneumonia of the British Society for Antimicrobial Chemotherapy. J Antimicrob Chemother. (2008) 62:5–34. doi: 10.1093/jac/dkn162, 18445577 PMC7110234

[131] Kalil AC Metersky ML Klompas M Muscedere J Sweeney DA Palmer LB . Management of Adults with Hospital-acquired and Ventilator-associated Pneumonia: 2016 clinical practice guidelines by the Infectious Diseases Society of America and the American Thoracic Society. Clin Infect Dis. (2016) 63:e61–e111. doi: 10.1093/cid/ciw353, 27418577 PMC4981759

[132] Rotstein C Evans G Born A Grossman R Light RB Magder S . Clinical practice guidelines for hospital-acquired pneumonia and ventilator-associated pneumonia in adults. Can J Infect Dis Med Microbiol. (2008) 19:19–53. doi: 10.1155/2008/593289, 19145262 PMC2610276

[133] Torres A Niederman MS Chastre J Ewig S Fernandez-Vandellos P Hanberger H . International ERS/ESICM/ESCMID/ALAT guidelines for the management of hospital-acquired pneumonia and ventilator-associated pneumonia. Eur Respir J. (2017) 50:1700582. doi: 10.1183/13993003.00582-2017 28890434

[134] European Centre for Disease Prevention and Control. European Surveillance of Healthcare- Associated Infections in Intensive Care Units – HAI-Net ICU Protocol Stockholm: ECDC; (2015). Available online at: https://www.ecdc.europa.eu/sites/default/files/media/en/publications/Publications/healthcare-associated-infections-HAI-ICU-protocol.pdf (Accessed September 1, 2025).

[135] Rello J Ausina V Castella J Net A Prats G. Nosocomial respiratory tract infections in multiple trauma patients. Influence of level of consciousness with implications for therapy. Chest. (1992) 102:525–9. doi: 10.1378/chest.102.2.525, 1643942

[136] Cook A Norwood S Berne J. Ventilator-associated pneumonia is more common and of less consequence in trauma patients compared with other critically ill patients. J Trauma. (2010) 69:1083–91. doi: 10.1097/TA.0b013e3181f9fb51, 21068613

[137] Antonelli M Moro ML Capelli O De Blasi RA D'Errico RR Conti G . Risk factors for early onset pneumonia in trauma patients. Chest. (1994) 105:224–8. doi: 10.1378/chest.105.1.224, 8275735

[138] Rello J Ollendorf DA Oster G Vera-Llonch M Bellm L Redman R . Epidemiology and outcomes of ventilator-associated pneumonia in a large US database. Chest. (2002) 122:2115–21. doi: 10.1378/chest.122.6.2115, 12475855

[139] Leonard KL Borst GM Davies SW Coogan M Waibel BH Poulin NR . Ventilator-associated pneumonia in trauma patients: different criteria, different rates. Surg Infect. (2016) 17:363–8. doi: 10.1089/sur.2014.076, 26938612

[140] Croce MA Fabian TC Waddle-Smith L Maxwell RA. Identification of early predictors for post-traumatic pneumonia. Am Surg. (2001) 67:105–10. doi: 10.1177/000313480106700201, 11243529

[141] World Health Organization. Global Antimicrobial Resistance and Use Surveillance System (GLASS) Report 2022 (2022). Available online at: https://www.who.int/publications/i/item/9789240062702 (Accessed September 1, 2025).

[142] Rai I Stephen AH Lu Q Heffernan DS. Impact of multi-drug-resistant pneumonia on outcomes of critically ill trauma patients. Surg Infect. (2020) 21:422–7. doi: 10.1089/sur.2019.240, 31895670 PMC7247025

[143] Cucci M Wooten C Fowler M Mallat A Hieb N Mullen C. Incidence and risk factors associated with multi-drug–resistant pathogens in a critically ill trauma population: a retrospective cohort study. Surg Infect. (2020) 21:15–22. doi: 10.1089/sur.2019.031, 31210580

[144] Torres A Lee N Cilloniz C Vila J Van der Eerden M. Laboratory diagnosis of pneumonia in the molecular age. Eur Respir J. (2016) 48:1764–78. doi: 10.1183/13993003.01144-2016, 27811073

[145] Younis HA Eisa TAE Nasser M Sahib TM Noor AA Alyasiri OM . A systematic review and meta-analysis of artificial intelligence tools in medicine and healthcare: applications, considerations, limitations, motivation and challenges. Diagnostics. (2024) 14:109. doi: 10.3390/diagnostics14010109, 38201418 PMC10802884

[146] Chumbita M Cillóniz C Puerta-Alcalde P Moreno-García E Sanjuan G Garcia-Pouton N . Can artificial intelligence improve the Management of Pneumonia. J Clin Med. (2020) 9:248. doi: 10.3390/jcm9010248, 31963480 PMC7019351

[147] Stephen O Sain M Maduh UJ Jeong D-U. An efficient deep learning approach to pneumonia classification in healthcare. J Healthc Eng. (2019) 2019:1–7. doi: 10.1155/2019/4180949, 31049186 PMC6458916

[148] Heckerling PS Gerber BS Tape TG Wigton RS. Prediction of community-acquired pneumonia using artificial neural networks. Med Decis Mak. (2003) 23:112–21. doi: 10.1177/0272989X03251247, 12693873

[149] Giang C Calvert J Rahmani K Barnes G Siefkas A Green-Saxena A . Predicting ventilator-associated pneumonia with machine learning. Medicine (Baltimore). (2021) 100:e26246. doi: 10.1097/MD.0000000000026246 34115013 PMC8202554

[150] Liang Y Zhu C Tian C Lin Q Li Z Li Z . Early prediction of ventilator-associated pneumonia in critical care patients: a machine learning model. BMC Pulm Med. (2022) 22:250. doi: 10.1186/s12890-022-02031-w 35752818 PMC9233772

[151] Frondelius T Atkova I Miettunen J Rello J Vesty G Chew HSJ . Early prediction of ventilator-associated pneumonia with machine learning models: a systematic review and meta-analysis of prediction model performance✰. Eur J Intern Med. (2024) 121:76–87. doi: 10.1016/j.ejim.2023.11.009, 37981529

[152] Hwang EJ Nam JG Lim WH Park SJ Jeong YS Kang JH . Deep learning for chest radiograph diagnosis in the emergency department. Radiology. (2019) 293:573–80. doi: 10.1148/radiol.2019191225 31638490

[153] van de Sande D van Genderen ME Huiskens J Gommers D van Bommel J. Moving from bytes to bedside: a systematic review on the use of artificial intelligence in the intensive care unit. Intensive Care Med. (2021) 47:750–60. doi: 10.1007/s00134-021-06446-7, 34089064 PMC8178026

[154] Becker J Decker JA Römmele C Kahn M Messmann H Wehler M . Artificial intelligence-based detection of pneumonia in chest radiographs. Diagnostics (Basel). (2022) 12:1465. doi: 10.3390/diagnostics12061465 35741276 PMC9221818

[155] Geng X Wu H Liu C Qi L Ballah AK Che W . Construction and validation of a predictive model of pneumonia for ICU patients with traumatic brain injury (TBI). Neurosurg Rev. (2023) 46:308. doi: 10.1007/s10143-023-02208-9, 37985473

[156] Kim J-H Chung K-M Lee J-J Choi H-J Kwon Y-S. Predictive modeling and integrated risk assessment of postoperative mortality and pneumonia in traumatic brain injury patients through clustering and machine learning: retrospective study. Biomedicines. (2023) 11:2880. doi: 10.3390/biomedicines11112880 38001880 PMC10669264

[157] Ashrafi N Abdollahi A Alaei K Pishgar M. Enhanced prediction of ventilator-associated pneumonia in patients with traumatic brain injury using advanced machine learning techniques. Sci Rep. (2025) 15:11363. doi: 10.1038/s41598-025-95779-0 40175458 PMC11965472

[158] Li S Feng Q Wang J Wu B Qiu W Zhuang Y . A machine learning model based on CT imaging metrics and clinical features to predict the risk of hospital-acquired pneumonia after traumatic brain injury. Infect Drug Resist. (2024) 17:3863–77. doi: 10.2147/IDR.S473825, 39253609 PMC11382661

[159] Bradley M Dente C Khatri V Schobel S Lisboa F Shi A . Advanced modeling to predict pneumonia in combat trauma patients. World J Surg. (2020) 44:2255–62. doi: 10.1007/s00268-019-05294-3, 31748888

